# The effect of counterfactual treatment on the consistency between ethnic minority values and value-expressive behavioral intentions

**DOI:** 10.3389/fpsyg.2025.1581564

**Published:** 2025-12-05

**Authors:** Yanli Yang

**Affiliations:** School of Marxism, North Minzu University, Yinchuan, China

**Keywords:** ethnic-minority-value, counterfactual thinking, value–behavior consistency, behavioral intentions, ethnicity

## Abstract

**Background:**

Consistency in values and behaviors has always been controversial in academia, particularly under specific situations. However, little is known about how situations affect the consistency of minority ethnic values with their value-expressive behaviors (reflecting the strength of an internal value characterized by the importance of ethnicity). Counterfactual thinking has been established to have a significant impact on behavioral change and performance improvement. Therefore, based on the theory of value–behavior consistency and the operational definition of minority ethnic values and counterfactual treatment, the study aimed to examine how counterfactual thinking affects the consistency between minority ethnic values and value-expressive behaviors.

**Methods:**

Using convenience sampling, 251 Chinese ethnic minority college students completed the Chinese Minority Ethnic Values Questionnaire (CMEVQ) to evaluate levels of minority ethnic values; 27% with the highest and lowest levels (total: 135) were divided into “ethnic closeness” and “ethnic alienation” groups. Subsequently, we selected five typical ethnic behavioral descriptions from the Chinese Minority Ethnic Value-Expressive Behavior Questionnaire (CMEVEBQ) and altered them to factual, counterfactual, and empirical thinking materials. Finally, both groups, as counterfactual priming groups, completed a pre-test (factual thinking baseline) and post-test (counterfactual thinking priming) using a sequential priming paradigm, which involved making choices regarding behavioral intention ratings and priming judgment; they, as empirical priming groups, did the similar quasi-experimental procedures 3 months later with an empirical thinking tasks included in the post-test.

**Results:**

The results indicated that (1) counterfactual treatment affect ethnic minorities in making choices regarding ethnic value-expressive behavioral intentions (promoting and inhibiting them); (2) in counterfactual treatment, ethnic minorities express their ethnic behavioral intentions in ethnic value-consistent directions; and (3) in counterfactual treatment, minorities with different levels of ethnic-minority-value have different levels of consistency in terms of ethnic values and value-expressive behavioral intentions.

**Discussion:**

Our findings indicate that ethnicity can protect and motivate ethnic minorities to maintain consistency between minority ethnic values and value-expressive behavioral intentions, which have important theoretical and practical implications for the value theory. This provides empirical evidence for enhancing national cohesion in forging a strong sense of community for the Chinese nation.

## 1 Introduction

Behaving according to one’s values or expressing values through behaviors can help people obtain a sense of self-consistency (Rokeach, 1973) and achieve their goals and aspirations (Bardi and Schwartz, 2003). People who sense consistency between their values and behaviors tend to experience positive feelings (Schwartz, 2007) and have high levels of self-esteem, self-fulfillment, and well-being (Rokeach, 1973; Sagiv and Schwartz, 2000; Bardi and Schwartz, 2003). This implies that people should express values that are important to them and act congruent with them. However, the behavioral choices consistent with values may be influenced by various factors unless they are self-consciously motivated by internal values (Baumeister and Twenge, 2002). Minority ethnic value is an internalized value characterized by minority ethnic members’ self-perception of the importance of ethnicity (Verkuyten, 2005; Yang et al., 2019b). It is also a subjective criterion for ethnic members to select and evaluate matters relevant to their ethnic group and protects group members against external pressure (Gelfand et al., 2011). However, due to the advancement of globalization and increasing contact with modern mainstream cultures, the “new” minority ethnic generation is more unaware of intergroup and intragroup differences than the “old” generation. Consequently, ethnic minority values are increasingly being challenged. Verkuyten (2005) suggested that the fading cultural differences may cause members to emphasize their ethnic belonging, particularly for the most acculturated minority individuals. The self-construal and norm activation theories (Schwartz, 1994) imply that social norms are internalized by members of minority ethnic groups, effectively promoting the related behavior once activated. Less attention has been devoted, however, to the processes through which values affect behavior among such a population (Roccas and Sagiv, 2017). Whether the reflections of minority ethnic values on ethnic group members’ actions and evaluation of events are congruent has remained unclear. The main goal of the current research is to assess the consistency in minority ethnic value and behavior from a perspective of counterfactual treatment.

### 1.1 Consistency in value and behavior

Consistency in values and behaviors has always been controversial in academia. Values are by nature socially positive (Rokeach, 1973) and relative stable across specific situations (Schwartz, 2014) and contexts (Schwartz, 1992). Researchers have suggested that the value–behavior relationship, despite potentially having strong situational constraints, is stronger at higher and weaker at lower levels of the relative priority of a specific value (Lake et al., 2024; Lee et al., 2022; Schwartz, 1992). Konty (2002) argued that value stability occurs only in similar specific environments rather than in all situations. When “reality” (i.e., situations) intervenes, individuals cannot always make the optimal choice that conform to their values in terms of its pressure (Shoda, 1999). People may temporarily overlook their own important values and reduce the corresponding behaviors vulnerable to strong situational constraints and threats, such as ecological, financial, institutional, time, choice-restricting, and environmental ones (Darley and Batson, 1973; Hamilton et al., 2019). Sagiv and Roccas (2021) examined the processes through which values lead to behavior and presented a conceptual model containing three organizing principles leading to minimal consistency between values and behavior: accessibility, interpretation, and control. The normative influence on behavioral expression in a value-consistent direction varies across a society’s culture (Elster and Gelfand, 2021), normative levels of values and behaviors (Bardi and Schwartz, 2003; Roccas and Sagiv, 2010), and the importance distribution of a value (Lee et al., 2022). Some researchers have attempted in experimental studies and employed a variety of priming tasks, raising the accessibility of values, to reveal consistent associations between values and behavior. (e.g., Verplanken and Holland, 2002; Maio et al., 2009b; Sagiv et al., 2011; Amit and Sagiv, 2013; Skimina et al., 2019). This is still a small body of research, however.

Few studies have assessed the consistency between values and behavior, likely owing to two issues. The first issue is that, in practice, behavior is particularly difficult to measure due to the variability caused by specific situational factors (Verkuyten, 2005; Maio, 2010). Researchers examined the link between changes in behavioral intentions and subsequent changes in actual behavior, drawing on experimental evidence (Gollwitzer and Sheeran, 2006; Webb and Sheeran, 2006). The result revealed that behavioral intention reflected an individual’s likelihood of performing a certain behavior in the future and was a direct indicator predicting behavior (Lewis et al., 2007). It is measured using reaction time to achieve better expected validity in predicting behavior than with self-reports (Peterson, 2005). This is mainly related to accessibility of information (Fan and Zhang, 2007). Accessibility reflects the strength of the connection between the psychological representation and decision-making behavior using reaction time (Cooke and Sheeran, 2013). Bassili (1995) proposed that high accessibility could increase the explanatory capacity of behavioral intention; in other words, shorter reaction time of behavioral intention judgement indicates faster speed and higher predictive validity of behavioral intention. In addition, typical examples of values, such as value-expressive behaviors (Verplanken and Holland, 2002; Hanel et al., 2017), have been highlighted to bridge the gap between values and behaviors and promote behaviors congruent with values (Lord et al., 1991; Roccas and Sagiv, 2017; Hanel et al., 2018). Schwartz and Butenko (2014) established 18 of 19 narrowly defined values correlated more positively with its corresponding value-expressive behaviors than with any other behaviors. Yang et al. (2019a) applied Schwartz’s refined value theory to assess the relationship between value and value-expressive behaviors among Chinese minorities, a multicultural population, and found that 13 of 19 universal values correlated most strongly with behaviors in value-consistent directions. Schwartz et al. (2017) further validated these findings with samples across various countries. The second issue lies in the lack of relatively general and consistent analytical approaches for measuring the consistency of relationships in academia (McBroom and Reed, 1992). Correlation is the most widely applied approach, and the correlation coefficient (r) is the main indicator for measuring consistency. Bardi and Schwartz (2003) suggested that value–behavior relationships were consistent for values that had stronger correlations with behaviors expressing them than with other behaviors. The aforementioned suggestion has been extensively used in research on value–behavior relations (Bardi and Schwartz, 2003; Schwartz and Butenko, 2014; Schwartz et al., 2017; Yang et al., 2019a,b). Additionally, Marks et al. (2014) defined consistency as behaving in a reliable manner across identical or similar situations; put differently, consistency refers to behaving in a similar or identical manner in response to repeated instances of a specific stimulus (or class of stimuli). They used a test–retest reliability coefficient (r_b, also known as r_xx) to represent consistency; that is, the correlation between two blocks of identical trials. We reviewed previous research and proposed that value–behavior consistency refers to the similarity between an individual’s behaviors and its leading values; in other words, when an individual’s behavioral performance conform to the motivational goal of their leading values, the values and behaviors are considered consistent. We wish to use the two above-mentioned analytical approaches to assess the consistency of value–behavior relations.

### 1.2 Counterfactual thinking

Although the impact of situational factors on the value–behavior relationship has achieved wide recognition and has been cited in many studies (Hitlin and Piliavin, 2004; Lönnqvist et al., 2006), empirical studies on this relationship are scarce mainly due to the selection of measurement indicators for situations. Research has shown that counterfactual thinking Counterfactual thinking has been established to serve a behavior regulating function, involving behavior change and performance improvement (e.g., Smallman and Roese, 2009). Counterfactual thoughts refer to mental simulations regarding how things could have turned out differently (Smallman and Roese, 2009; Xie and Beck, 2022). They may happen after good events (e.g., lucky wins and near misses) and bad events (e.g., failures and tragic accidents), but they do so more often after bad events (e.g., Sanna and Turley, 1996). Counterfactuals are typically expressed through thoughts phrased as “Would I have had a better outcome if I had chosen a different option (i.e., “if only…, then…”)? For example, “If I had not been driving so fast, I would not have been in an accident”; “If only I’d studied harder, I’d have passed the exam.” Such self-referent upward counterfactuals, i.e., thoughts about how things could have been better, may lead to ameliorative action in the future, but at an affective cost—they can lead people to experience negative emotions, such as self-blame, regret, and guilt (e.g., Roese, 1997; Mandel and Dhami, 2005; Epstude and Roese, 2008; Broomhall and Phillips, 2018). The notion that negative affect is an important ingredient for counterfactual activation is consistent with a functional approach to counterfactual thinking (e.g., Markman et al., 2008; for details see also Roese and Hur, 1997) and constitutes a signal to the problems or danger requiring corrective action (e.g., Schwarz, 1990; Taylor, 1991); this may cause greater psychological stress than not using the thought due to the associated negative emotions (Davis and Lehman, 1995; Roese, 1997; Epstude and Roese, 2008). Markman et al. (2008) concluded that upward counterfactual due to emotional stress evoked by failure induces greater effort and striving on a subsequent task. Blix et al. (2016) pointed to a possibility that people may not only develop post-traumatic stress disorder as a result of actual experiences but also via mental simulations of traumatic events that could have happened—mental simulations that were generated by upward counterfactual thinking. Moreover, Smallman and Roese (2009) conducted an experiment that activated counterfactual thinking and past experience using a sequential priming paradigm and demonstrated that counterfactual thinking promoted positive behavioral intention, which was driven by negative affect. They also suggested that, whether using Likert intention ratings or a sequential priming paradigm, there are two ways in which counterfactuals can influence behavior, a content-specific and a content-neutral pathway. Page and Colby (2003) outlined that the content-specific counterfactuals influenced behavior through behavioral intention. Hence, counterfactual thinking may play a positive or negative role (Gilbar and Hevroni, 2007). This study attempted to examine the role of content-specific counterfactuals as a situational factor in behavior change within the Chinese cultural context.

### 1.3 Ethnic minority value

Intergroup literature indicates that ingroup members prioritize ingroup (over outgroup) welfare in contexts of environmental threats and enhance cohesion in the form of ingroup bonding (Cheon and Hong, 2016). This ingroup bias may reduce stress, as social support buffers against the negative effects of threats (Frisch et al., 2014). These findings imply that ethnic minorities would engage in ethnic behaviors in minority-ethnic-value-consistent directions in stressful situations. The ingroup bias is ethnicity, which is of particular value for members of ethnic groups in self-construal and in identifying each other, allowing them to automatically categorize themselves and others into different groups due to a common imagined origin and cultures (Verkuyten, 2005). Yang et al. (2019b) used ethnic minority values and value-expressive behaviors of ethnic minorities to describe the common reflections and *a priori* behavior, respectively, that ethnic members use to indicate their importance of ethnicity; then, they identified four of six values correlated most strongly with value-expressive behaviors in value-consistent directions: minority ethnic exploration (MEE), involvement (MEIV), alienation (MEA), and inheritance (MEIH). Additionally, minority ethnic individuals with strong self-perceived ethnicity (ethnic minority values), that is the ethnic closeness group, are psychologically or practically close to their own minority ethnic groups. For example, they frequently seek opportunities to explore their ethnic knowledge subjectively, inherit their own ethnic culture in certain ways (e.g., ethnic festivals or written record), and protect their own ethnic interests to ensure they are not violated. Contrastingly, members of the ethnic alienation group have poor self-perception of the importance of ethnicity (minority ethnic values), are indifferent to ethnic matters of their own, and are psychologically or practically distant from their own minority ethnic groups. However, few studies have examined the relationship between minority ethnic values and behaviors, and the psychological mechanism by which situational factors influence this relationship remain unclear.

This study used a sequential priming paradigm and behavior-intention judgement tasks. Counterfactual thinking scenarios related to expressing minority ethnic values were employed as situational priming stimuli, and ethnic behavioral intention judgments were used as probing stimuli. We explored whether the primed situation affected the behavioral intentions expressing minority ethnic values of various levels. This study posed the following questions. First, can counterfactual treatment affect behavioral intentions expressing minority ethnic values? We proposed that counterfactual treatment would improve or inhibit behavioral intention choices of ethnic minorities in expressing ethnic values (Hypothesis 1). Second, some scholars have found that individuals who are self-consciously motivated by internal values make behavioral choices consistent with values despite the influence of various factors. Then, would a minority ethnic value–behavior relationship characterized by expressing ethnicity from the ethnic “inside” remain consistent? We proposed that ethnic minorities would express behavioral intentions in ethnic-value-consistent directions in counterfactual treatment (Hypothesis 2). Third, previous studies have suggested that the value–behavior relationship differ across various levels of a value. Therefore, can counterfactual treatment exert influence on consistency between minority ethnic values and value-expressive behavioral intentions? We proposed that counterfactual treatment would affect consistency between ethnic values and value-expressive behavioral intentions among minorities with various levels of ethnic minority values (Hypothesis 3).

## 2 Methods

### 2.1 Participants

Owing to the goal sample size of effect size association validity is determined via the calculation result of G*Power, considering two groups. We conducted independent samples *t*-tests using G*Power 3.1.9.2 (Faul et al., 2007) to allow detection of a medium effect (|*ρ*| = 0.50) with alpha at 0.05 and power of 0.80. This calculation detected a required sample size of 128 participants, meaning that the number of test group members for the final analysis in the current study needed to be greater than or equal to 128. Moreover, we established the two groups (groups of high and low levels of ethnic minority values, respectively) via the critical ratio grouping with a 27% cut-off (Nunnally and Bernstein, 1994), so as to maximize the sensitivity of *t*-tests for mean score differences across ability groups, consequently optimizing item discrimination analysis validity. As such, we aimed to recruit approximately 250 participants (54% test-group and 46% non-test-group participants). Finally, a convenience sampling method was adopted to recruit 251 minority ethnic college students from North Minzu University (see Attachment for a more detailed overview of the characteristics of the university and ethnic minorities at this university). All participants completed the Chinese Minority Ethnic Value Questionnaire (CMEVQ) via a Chinese online survey platform, as this scale has been shown to represent the important ethnic characteristics of ethnic minorities (Yang et al., 2019b). We used the average scores of the four of six dimensions of the CMEVQ (Minority Ethnic Consciousness, Involvement, Exploration, and Inheritance) to evaluate the level of minority ethnic values among the participants. The average scores were arranged in descending order. The top 27% of the scores were in the high-ethnic-value group (*M* = 5.20, *SD* = 0.36), labeled as the ethnic closeness group. The bottom 27% of the scores were classified as the low-ethnic-value group (*M* = 3.69, *SD* = 0.54), labeled as the ethnic alienation group. Finally, 67 participants were categorized into the ethnic closeness group (*M*_age_ = 19.64, *SD* = 1.20) and 68 participants into the ethnic alienation group (*M*_age_ = 20.16, *SD* = 1.79). All participants were right-handed, had normal or corrected-to-normal vision, had no previous participation in relevant trials. They had no significant difference in text-reading speed, measured in seconds [*M*_closeness_ = 169.10, *SD* = 8.40; *M*_alienation_ = 170.68, *SD* = 4.93; *t*_(106)_ = 1.324, *p* = 0.188]. Each participant was paid between 10 and 15 yuan after the end of the test. Participant characteristics are presented in Table 1.

**Table 1:** Demographic data (*n* = 135).

Variable	Composition	Frequency			
		Closeness (*n*)	Closeness (%)	Alienation (*n*)	Alienation (%)
Ethnic	Hui (Hui)	14	20.9	7	10.3
	Uighur (Uighur)	10	14.9	6	8.8
	Mongol (Zhuang)	9	13.4	8	11.8
	Dong (Dong)	8	11.9	5	7.4
	Tibetan (Tibetan)	7	10.4	1	1.5
	Tujia (Tujia)	3	4.5	8	11.8
	Miao (Miao)	3	4.5	7	10.3
	Bai (Bai)	2	3.0		
	Yao (Qiang)	2	3.0	4	5.9
	Buyi (Kazak)	2	3.0	3	4.4
	Man (Man)	1	1.5	4	5.9
	She (She)	1	1.5	2	2.9
	Hani (Hani)	1	1.5	4	5.9
	Lisu (Dai)	1	1.5	3	4.4
	Gelao (Tu)	1	1.5	3	4.4
	Lahu (Yi)	1	1.5	2	2.9
	Mulao (Mulao)	1	1.5	1	1.5
Gender	Female	37	55.2	41	60.3
	Male	30	44.8	27	39.7
Parental ethnicity	Same	52	77.6	37	54.4
	Different	15	22.4	31	45.6
Source area	Rural	35	52.2	18	26.5
	Urban	17	25.4	21	30.9
	Town	15	22.4	29	42.6
Residence status	Gathered-residing	53	79.1	25	63.2
	Scattered-residing	14	20.9	43	36.8

In parentheses is the composition of participants in ethnic alienation group.

### 2.2 Test materials

#### 2.2.1 CMEVQ and CMEVEBQ

CMEVQ and Chinese Minority Ethnic Value-Expressive Behavior Questionnaire (CMEVEBQ) are well-matched and mutually independent ethnic questionnaires. These scales were developed by Yang et al. (2019b) with a sample of 39 of 55 Chinese ethnic minorities, who were mainly college students. Each scale comprises 18 items in six shared dimensions: Minority Ethnic Consciousness (MEC), Involvement (MEIV), Alienation (MEA), Exploration (MEE), Inheritance (MEIH), and Mastery (MEM). The two scales are sufficiently heterogeneous to cover the importance and common contents of minority ethnic cultures and behavioral manifestations.

Each item in the CMEVQ has a short, gender-matched, verbal portrait of diverse minority ethnic member, each implying a minority ethnic value held by the member. Respondents’ minority ethnic values are derived from their reports of how similar the member is to themselves in each portrait on a six-point Likert scale (1 = not like me at all; 6 = very much like me). For instance, “It is important to them to honor their own ethnic diverse taboos” describes an ethnic minority member who emphasizes MEC. Higher average scores in the four dimensions indicate a higher level of minority ethnic values. The internal reliability of the overall scale and the six subscales in this study ranged from 0.66 to 0.83.

The behaviors in the CMEVEBQ were generated specifically to match the value dimensions of the CMEVQ. Respondents report how often they engage in each behavior relative to opportunities to do so on a five-point Likert scale (0 = never; 4 = always). The behavior items match the corresponding ethnic values. For instance, “Celebrate your own ethnic festivals with families and friends” matches MEC, “Spread information about your ethnic culture” matches MEE, and “Not care whether you self-perceive your ethnicity” matches MEA. In the original study, the overall scale and the three behavior subscales (MEC, MEE, and MEIH) to be employed in the present research demonstrated reliabilities of 0.81, 0.72, 0.74, and 0.77, respectively.

#### 2.2.2 Think materials

We selected five item contents, namely the typical ethnic behavioral descriptions (as described below) from 3 of 6 dimensions of the CMEVEBQ, i.e., MEC, MEE, and MEIH, because their internal consistency coefficients of the three dimensions were among the top three in the original study. We altered these behavioral descriptions to the corresponding negative consequences, namely ethnic alienation intentions, as factual thinking materials. Subsequently, we gave the five factual thinking materials conditional propositions that were contrary to the negative consequences that had already occurred in the imagination. This created the upward counterfactual thinking materials containing an antecedent (if) and consequent (then). We also provided the five typical ethnic behavioral descriptions with their corresponding empirical thinking descriptions. The following factual, counterfactual, and empirical thinking materials were used:

Example 1 (MEC):

*Please imagine that you were asked by a classmate about your ethnic customs and habits, and you cannot answer because you do not know anything.*

*If I can learn more about my ethnic culture in my daily life, then I can introduce related customs and habits to my classmates.*

*I learn more about my ethnic culture whenever I have the opportunity in daily life.*

Example 2 (MEIH):

*Please imagine that you feel the unique culture of your ethnic group gradually disappearing, and you do nothing and do not care about it.*

*If all members of my ethnic group, starting with me, can preserve our unique customs and habits in daily life, then our ethnic culture will be passed down.*

*I can preserve the unique customs and habits of my ethnic group in daily life.*

Example 3 (MEE): *Please imagine that you heard a classmate misunderstand your ethnic culture, and you know the facts but are unwilling to explain it.*

*If I heard a classmate misunderstand my ethnic culture, then I will promptly explain the facts to reduce misunderstandings.*

*I will introduce others about my ethnic culture to reduce their misunderstandings about my ethnic culture.*

Example 4 (MEC):

*Please imagine that you have not felt your minority ethnic identity for a long time.*

*If I exhibit the characteristics of my own ethnic group in my daily life, then I will have a stronger sense of ethnic identity.*

*I exhibit the characteristics of my own ethnic group in my daily life to maintain a sense of ethnic identity.*

Example 5 (MEE):

*Please imagine that some minority ethnic students were excited to find unique items of their ethnic group, such as handicrafts and clothing, in an exhibition of ethnic products; however, you could not identify any items of your ethnic group.*

*If I pay enough attention to my ethnic culture in daily life, including accumulating relevant knowledge and participating in relevant activities, then I will not be ignorant of the unique items of* my ethnic group.

*I exhibit the characteristics of my own ethnic group in my daily life to maintain a sense of ethnic identity.*

#### 2.2.3 Behavior-intention judgement task materials

This study used two facets of behavioral intention. One was the degree of agreement that the respondent would perform ethnic behaviors in the future, and the other was the possibility of the respondent performing ethnic behaviors in the future (Conner and Mcmillan, 1999). Respondents rated their ethnic behavioral intentions on a six-point Likert scale (1 = nearly impossible; 6 = very likely), with higher scores indicating more active engagement in the corresponding ethnic behavioral intentions.

### 2.3 Study design

The test used a 2 (type of ethnic minority value: ethnic closeness, ethnic alienation) × 2 (priming: counterfactual thinking and empirical thinking) mixed design, with the first factor as a between-subject and the last as a within-subject factor. Two groups of participants with high-low levels of ethnic minority values completed the counterfactual thinking tasks first, i.e., the counterfactual priming condition, followed by the empirical thinking tasks 3 months later, i.e., the empirical priming condition. The dependent variables were the rating score and reaction time for ethnic behavioral intention.

### 2.4 Test tasks and procedures

**Figure 1:**
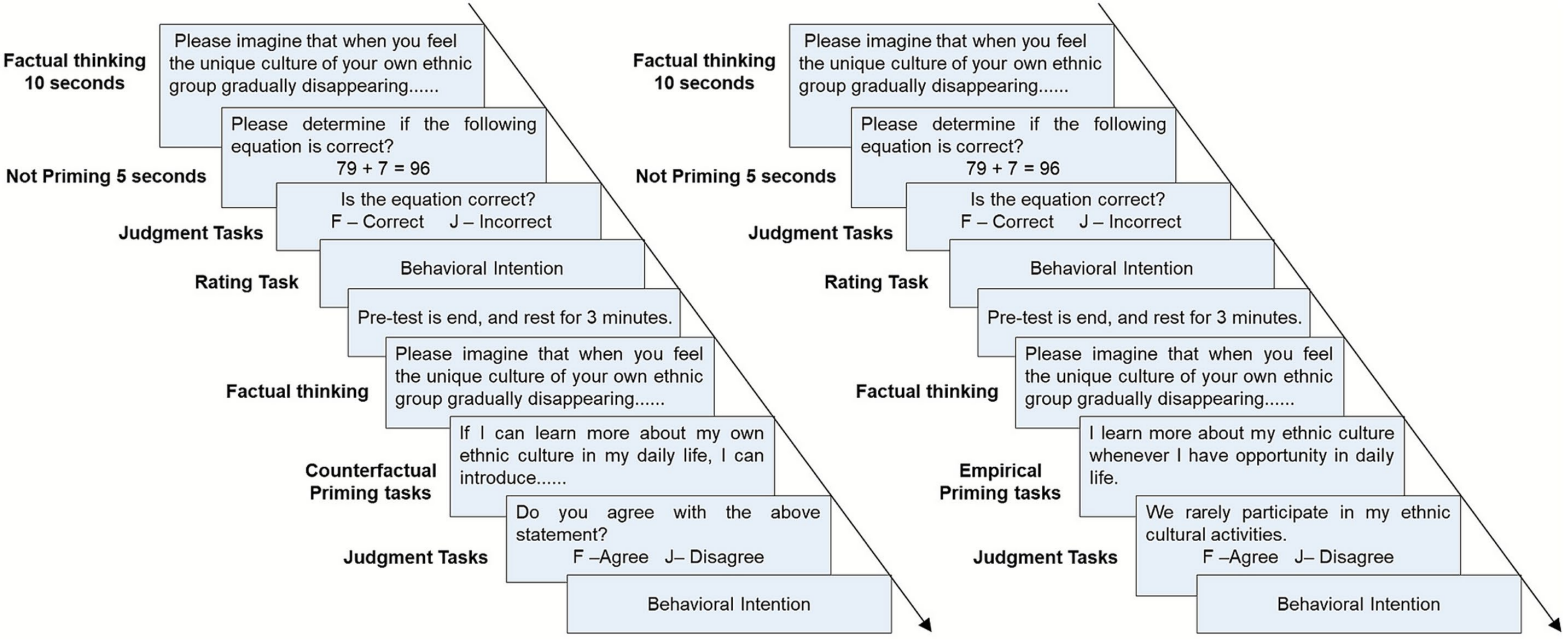
Flow chart of single trial.

E-prime 2.0 software was used to compile the test program. Two blocks included pre- and post-test tasks and were used for the entire quasi-experimental work. A pre-test measured participants’ factual thinking baseline, whereas a post-test was used to measure counterfactual or empirical thinking priming. The block consisted of ten trials, each comprising two tasks: priming judgement and behavioral intention rating. The procedure is illustrated in Figure 1.

For the pre-test task, the factual thinking material was presented on a screen for 10 s in each trial to ask participants to imagine the situation happening to themselves. Subsequently, an interference task comprising simple addition and subtraction operations was presented to prevent participants from spontaneously having counterfactual thinking to influence the study results, as spontaneous counterfactual thinking has a greater impact on an individual’s persistence than induced counterfactual thinking (Tal-Or et al., 2006). The participants were then asked to judge whether the equation was correct in 5 s by pressing the “F” key for correct and “J” key for incorrect. Following this, two rating tasks of ethnic value-expressive behavioral intention were presented, and E-prime 2.0 was used to record the participants’ rating scores and reaction time from the presentation of the behavioral intention description until the participants responded.

After the pre-test, 3 min of soft music was played in the laboratory to allow the participants to rest sufficiently. All participants were then asked to complete the post-test.

The post-test tasks are the corresponding priming tasks. Firstly, the same factual thinking material used in the pre-test task was presented on the screen for 10 s to ask participants to imagine the situation happening to themselves. Subsequently, the tasks under the counterfactual priming condition, which provided typical counterfactual thinking descriptions corresponding to the factual thinking materials, was presented on the screen for 5 s. Two sentence structures containing an antecedent (if) or a consequent (then) were randomly presented to exclude the influence of specific grammar on the test results. Participants then judged whether they agreed with the above description, that is, whether the description would change the negative consequences that occurred in the factual thinking situations, by pressing the same keys as described above. The priming task under the empirical condition was presented with empirical thinking descriptions corresponding to the factual thinking materials. To prevent participants from blindly responding with a “Yes” in the judgment task without thinking about the priming information, a random ethnic behavioral description with a different frequency was presented after the empirical priming task. Participants were then asked to judge whether the description was in accordance with their daily ethnic behaviors, by pressing the “F” key to agree and the “J” key to disagree. Finally, the participants of two conditions repeated the same ratings of behavioral intentions used in the pre-test, with software recording the participants’ rating scores and reaction times from the presentation of the behavioral intention description until the participants responded.

## 3 Results

### 3.1 Manipulation check

Prior to hypothesis testing, we performed an outlier analysis and checked extreme reaction time exceeding 2 standard deviations in each trial (Ratcliff, 1992). All the data entered the final processing and analysis with IBM SPSS 26.0.

### 3.2 Behavioral intention difference before and after priming treatment

Table 2 shows the descriptive statistical results and *t*-tests results for the rating scores and reaction times of ethnic behavioral intention between participants in the closeness and alienation groups, during the pre- and post-tests under different priming conditions. A paired samples *t*-test was conducted separately on behavioral intention scores and reaction times for the two groups of participants in the two testing tasks. In the counterfactual priming, the post-test behavioral intention score of the ethnic closeness group in the MEC, MEE, and MEIH dimensions was significantly higher than the corresponding pre-test score, and the same results emerged in the first two dimensions for the ethnic alienation group. In the empirical priming, only the post-test behavioral intention score of the ethnic alienation group in the MEC dimension was significantly higher than the corresponding pre-test score. Additionally, the reaction time in the post-test was significantly shorter than that in the pre-test for the two groups under the two priming conditions. The results demonstrated that both groups rated behavioral intention higher and responded faster after counterfactual priming than at the baseline level.

**Table 2:** Descriptive statistics and *t*-tests of independent variables (*n* = 135).

Priming	Dependent variables	Dimension	Mean		Standard Deviation		*t*-test		
			Pre-test	Pro-test	Pre-test	Pro-test	PST	IST	
								Pre-test	Pro-test
Counterfactual 1 (*n* = 67) 2 (*n* = 68)	RS	MEC	4.80	4.98	0.76	0.74	−2.45^*^	3.45^**^	3.99^***^
			4.35	4.51	0.75	0.61	−2.81^**^		
		MEE	4.87	5.07	0.73	0.77	−2.19^*^	2.92^**^	3.20^**^
			4.49	4.62	0.77	0.85	−2.05^*^		
		MEIH	4.94	5.19	1.01	0.73	−2.13^*^	4.04^***^	5.97^***^
			4.31	4.40	0.81	0.82	−1.27		
	RT (ms)	MEC	5156.56	2482.55	1557.83	1084.03	17.05^***^	1.54	−1.08
			4821.68	2664.35	876.02	850.35	16.34^***^		
		MEE	3868.62	2296.48	1504.85	1081.20	9.34^***^	1.23	0.43
			3595.49	2222.53	1039.45	908.52	11.17^***^		
		MEIH	4554.06	2331.02	1591.62	1203.86	10.38^***^	−0.61	−1.25
			4707.14	2594.37	1318.67	1251.73	11.14^***^		
Empirical 1 (*n* = 67) 2 (*n* = 68)	RS	MEC	4.83	4.89	0.80	0.73	−0.79	3.65^***^	3.36^**^
			4.25	4.41	1.04	0.93	−2.17^*^		
		MEE	4.79	4.88	0.92	0.79	−0.92	2.73^**^	3.05^**^
			4.36	4.43	0.89	0.93	−1.05		
		MEIH	4.99	4.96	0.85	0.76	0.48	4.33^***^	4.60^***^
			4.29	4.26	1.02	0.98	0.33		
	RT (ms)	MEC	5168.70	2513.81	1773.26	1031.29	14.04^***^	1.62	−0.51
			4766.03	2597.82	955.76	875.43	15.55^***^		
		MEE	3580.34	2101.05	1526.82	1078.43	8.18^**^	0.58	−0.33
			3448.86	2158.38	1057.51	888.66	9.49^***^		
		MEIH	4610.44	2204.96	1467.32	1184.00	11.19^***^	0.25	−1.7
			4551.89	2548.83	1234.78	1153.64	11.16^***^		

*p < 0.05, **p < 0.01, ***p < 0.001 (two-tailed). 1 = ethnic closeness group, 2 = ethnic alienation group. RS, Rating Score of Behavioral Intention. RT, Reaction Time. PST, Paired samples *t*-test. IST, Independent samples *t*-test. MEC, minority ethnic consciousness; MEE, minority ethnic exploration; MEIH, minority ethnic inheritance.

### 3.3 Difference in behavioral intention between high and low value levels

We conducted an independent samples *t*-test on behavioral intention scores and reaction times between the ethnic closeness and alienation groups of participants in the pre-test and post-test under two priming conditions. The results in Table 2 revealed that the behavioral intention score of the ethnic closeness group was significantly higher than that of the ethnic alienation group in both tests and priming conditions; however, no significant difference was observed in the reaction time. This indicated that the counterfactual thinking led the ethnic closeness group to make more positive choices regarding ethnic behavioral intention than the ethnic alienation group, but not lead them to respond faster.

Due to the grouping of participants based on high-low levels of minority ethnic values and the priming conditions of counterfactual-empirical thinking, we conducted a mixed-factorial analysis of covariance (ANCOVA) to eliminate the effect of the two categorical independent factors on behavioral intention scores and reaction time in the post-test. We used the ethnic behavioral intention score and reaction time of three dimensions, respectively, in the pre-test as covariates, with the groupings of participants’ value-levels and priming tasks as two independent variables, and their corresponding rating score and reaction time in the post-test as the dependent variables. The results of variance homogeneity test showed that all the behavioral intention score [*F*_MEC (3, 266)_ = 0.74, *p* = 0.53; *F*_MEE (3, 266)_ = 0.76, *p* = 0.52; *F*_MEIH (3, 266)_ = 1.82, *p* = 0.14] and reaction time [*F*_MEC (1, 133)_ = 0.32, *p* = 0.82; *F*_MEE (1, 133)_ = 1.06, *p* = 0.37; *F*_MEC (1, 133)_ = 0.23, *p* = 0.88] met the condition of variance homogeneity. For the MEC dimension, a significant main effect of ethnic value grouping was observed on reaction time [*F*_(1, 266)_ = 5.96, *p* < 0.05, *η_p_*^2^ = 0.022], and the effect on behavioral intention was marginal [*F*_(1, 266)_ = 3.67, *p* = 0.06, *η_p_*^2^ = 0.014]. For the MEE dimension, a significant main effect appeared on the behavioral intension in ethnic value grouping [*F*_(1, 266)_ = 5.18, *p* < 0.05, *η_p_*^2^ = 0.019]. For the MEIH dimension, a significant main effect appeared on behavioral intention in both ethnic value grouping [*F*_(1, 266)_ = 18.44, *p* < 0.001, *η_p_*^2^ = 0.065] and priming condition [*F*_(1, 266)_ = 7.23, *p* < 0.01, *η_p_*^2^ = 0.026] and did on reaction time [*F*_(1, 266)_ = 4.36, *p* < 0.05, *η_p_*^2^ = 0.016] in only ethnic value grouping. Specifically, the rating scores of ethnic behavioral intention in three dimensions among the ethnic closeness group were higher than that among the ethnic alienation group. The reaction times of the ethnic closeness group in MEC and MEIH dimensions were significantly shorter than that of the ethnic alienation group. Moreover, participants’ rating score of ethnic behavioral intention in MEIH was significantly higher in the counterfactual than in the empirical priming. However, the two-way interaction between ethnic value grouping and priming condition was not statistically significant on the ethnic behavioral intention [*F*_MEC (1, 265)_ = 0.39, *p* = 0.53; *F*_MEE (1, 265)_ = 0.02, *p* = 0.89; *F*_MEIH (1, 265)_ = 1.03, *p* = 0.31] and reaction time [*F*_MEC (1, 265)_ = 0.06, *p* = 0.80; *F*_MEE (1, 265)_ = 0.19, *p* = 0.67; *F*_MEIH (1, 265)_ = 0.19, *p* = 0.66] in either of dimensions. Thus, the results indicating that the ethnic closeness group made more positive choices regarding ethnic behavioral intentions as compared to the ethnic alienation group were validated. The participates rated ethnic behavioral intention higher in the counterfactual than in the empirical priming. No significant interaction indicated that the effect of priming condition did not differ substantially as a function of an individual’s ethnic value level.

In addition, we examined differences in demographic variables that may have affected ethnic value levels, namely, family residence area (i.e., gathered or scattered, rural or urban) and parental ethnicity (i.e., same or different) between the two groups of participants shown in Table 1 using a chi-square test. The results revealed that the number of the ethnic closeness participants from minority-gathered areas (*x^2^* = 22.70, *p* < 0.001), rural areas (*x^2^* = 10.87, *p* < 0.01) or with parents of the same ethnic group (*x^2^* = 22.43, *p* < 0.001) was significantly higher than that from minority-scattered areas, non-rural areas or having parents of the different ethnic group. For the ethnic alienation group, the number of participants from minority-scattered areas (*x*^2^ = 4.77, *p* < 0.05) was significantly higher than that from minority-gathered areas, and no significant differences in the number of participants were observed in terms of their source areas and parental ethnicity.

### 3.4 Associations between ethnic minority values and behavioral intentions

Finally, we, respectively, conducted Pearson correlation tests to determine the link between the six factors of minority ethnic values and rating scores of ethnic behavioral intentions pre- and post-test in two priming conditions. On the one hand, we expected that the behavioral intentions, whose contents were selected from the MEC, MEE, and MEIH dimensions of the CMEVEBQ, would correlate more strongly with their expressive values than with the other three values (MEIV, MEA, and MEM in CMEVEBQ); on the other hand, we wished to explore the discrepancies of the six sets of minority ethnic value–behavior relationships across various levels of a value. As shown in Table 3, for the ethnic closeness group, MEE value correlated most strongly with its corresponding behavioral intentions in both the pre- (*p* = 0.08) and post-test (*p* = 0.09) and the counterfactual-empirical priming (*p*_s_ < 0.05); MEIH value correlated most strongly with its corresponding behavioral intentions in the pre-test of the counterfactual priming (*p* < 0.05) and in the post-test of the empirical priming (*p* < 0.05); the strongest correlation of MEC only emerged in the pre- (*p* = 0.11) and post-test (*p* < 0.05) of the empirical priming. Moreover, the correlation coefficients of MEIV increased in the post-test of the empirical priming compared to the counterfactual condition, with a significance (*p* < 0.01) appearing in the behavioral intentions of MEC and MEE and a marginal significance (*p* = 0.09) in the MEIH. All correlation coefficients increased in the post-test of the empirical priming compared to the counterfactual priming for the ethnic closeness group. For the ethnic alienation group, MEC value correlated most strongly with its corresponding behavioral intentions in the pre- (*p* < 0.05) and post-test (*p* = 0.09) of the empirical priming. MEIH value correlated most strongly with its corresponding behavioral intentions in the post-test of two priming conditions (*p*_counterfactual_ < 0.01, *p*_empirical_ < 0.05), but correlated most strongly with the behavioral intentions of MEC dimension in the pre-test (*p*_s_ < 0.05); the strongest correlation of MEE only emerged in the pre-test (*p* = 0.06) of the empirical priming. Furthermore, 10 of 12 value–behavior intention correlations of MEM were higher in the ethnic alienation group than in the ethnic closeness group, especially with a significance (*p* < 0.05) appearing in MEC and MEIH dimensions in the post-test of the counterfactual priming. This indicated that participants in the ethnic closeness group maintained more consistent minority ethnic value–behavior intention relationships than ones in the ethnic alienation group, but this trend was especially obvious in the empirical priming condition.

**Table 3:** Correlations between minority ethnic values and ethnic behavioral intentions.

Grouping	Priming	MEV	pre-test			pro-test		
			MEC_b_	MEE_b_	MEIH_b_	MEC_b_	MEE_b_	MEIH_b_
1 (*n* = 67)	Counterfactual	MEC	−0.12	−0.12	0.00	−0.16	−0.15	−0.17
		MEE	0.06	0.22^a^	0.21^a^	0.14	0.21^a^	0.15
		MEIV	0.13	0.10	0.05	0.21	−0.04	0.00
		MEA	−0.09	−0.21^a^	−0.09	−0.13	−0.13	−0.16
		MEIH	0.08	0.18	**0.26** ^*^	0.22^a^	0.18	0.09
		MEM	0.10	0.07	0.17	0.28^*^	0.03	0.04
	Empirical	MEC	0.20	0.06	0.18	**0.28** ^*^	0.15	0.20
		MEE	0.12	**0.26** ^*^	0.15	0.18	**0.27** ^*^	0.15
		MEIV	0.27^*^	0.18	0.13	0.32^**^	0.33^**^	0.21^a^
		MEA	−0.28^*^	−0.17	−0.08	−0.18	−0.16	−0.14
		MEIH	0.28^*^	0.17	0.20	0.23^a^	0.24^a^	**0.29** ^*^
		MEM	0.19	0.09	0.07	0.26^*^	0.14	0.21^a^
2 (*n* = 68)	Counterfactual	MEC	0.22^a^	0.25^*^	0.18	0.23^a^	0.18	0.41^**^
		MEE	0.27^*^	0.25^*^	0.16	0.21^a^	0.19	0.27^*^
		MEIV	0.01	0.13	0.14	−0.03	0.06	0.19
		MEA	0.00	−0.04	0.00	0.09	0.04	−0.15
		MEIH	0.28^*^	0.19	0.16	0.09	0.10	**0.33** ^**^
		MEM	0.20	0.16	0.27^*^	0.25^*^	0.20	0.31^*^
	Empirical	MEC	0.29*	0.14	0.11	**0.20** ^**a**^	0.14	0.12
		MEE	0.18	**0.23** ^**a**^	0.13	0.20^a^	0.17	0.08
		MEIV	0.18	0.08	0.21^a^	0.24^*^	0.09	0.19
		MEA	−0.11	−0.08	−0.17	−0.14	−0.09	−0.19
		MEIH	0.27^*^	0.13	0.17	0.19	0.14	0.24*
		MEM	0.24^a^	0.22^a^	0.19	0.25^*^	0.20	0.20

*p < 0.05, **p < 0.01 (two-tailed). The subscript b means the dimension of behavioral intention. Grouping: 1 = ethnic closeness group, 2 = ethnic alienation group. MEV, minority ethnic value; MEC, minority ethnic consciousness; MEE, minority ethnic exploration; MEIV, minority ethnic involvement; MEA, minority ethnic alienation; MEIH, minority ethnic inheritance; MEM, minority ethnic mastery. Bold values denote the strongest correlation coefficients within each variable set.

a *p* means a marginal significance.

To further verify minority ethnic value–behavior intention relations, we checked the predictive roles of six ethnic values to scores of ethnic behavioral intentions pre- and post-test under two priming conditions. Separately, with each of the rating scores of behavioral intentions (the pre-post test) in MEC, MEE, and MEIH as the target variable, the predictors entered in the multiple regression comprised the six ethnic values. As shown in Table 4, for the ethnic closeness group, the value of MEIH (*β* = 0.64) and MEE (*β* = 0.46) explained the most variance of its corresponding pre-test behavioral intention score, respectively, under the counterfactual and empirical priming; under the empirical priming, MEIH value (*β* = 0.27) most explained the post-test behavioral intention score of MEIH, and the most variance of the post-test behavioral intention score of both MEC (*β* = 0.37) and MEE (*β* = 0.38) appeared in the value of MEIV. For the ethnic alienation group, the value of MEE (*β* = 0.25) and MEC (*β* = 0.30) explained the most variance of its corresponding pre-test behavioral intention score, respectively, under the counterfactual and empirical priming; under the empirical priming, the most variance of the post-test behavioral intention score of MEIH was explained by its corresponding value (*β* = 0.24); the most variance of MEC behavioral intention was explained by the MEE (*β* = 0.30) and MEM (*β* = 0.24) values before and after the counterfactual priming, respectively; and the most variance of MEIH behavioral intention was explained by the MEE (*β* = 0.36) and MEC (*β* = 0.39) values before and after the counterfactual priming, respectively. The results, largely consistent with the correlation analyses, revealed that distinct ethnic value levels drove divergent ethnic motivations: the ethnic closeness group preferred to ethnic involvement by ethnic exploration and inheritance, whereas the alienation group showed a tendency to enhance ethnic consciousness via the same means.

**Table 4:** Regression of ethnic minority value factors on ethnic behavioral intentions (Only significant predictive Independent variables).

G	BI	P	DV	IV	USC		SC	*t*	CS		DW (U)	*R* ^2^
					B	SE	Beta		T	VIF		
1 (*n* = 67)	Pre-test	C	B-MEIH	(Constant)	1.38	1.67		0.82			2.38	0.07
				V-MEIH	0.64	0.3	0.26	2.14^**^	1	1		
		E	B-MEC	(Constant)	4.10	0.62		6.65^***^			0.40	0.14
				V-MEA	−0.21	0.10	−0.26	−2.24^*^	0.99	1.01		
				V-MEIH	0.25	0.12	0.25	2.15^*^	0.99	1.01		
			B-MEE	(Constant)	3.18	0.73		4.33^***^			1.29	0.14
				V-MEE	0.46	0.16	0.35	2.86^**^	0.89	1.12		
				V-MEA	−0.27	0.12	−0.28	−2.30^*^	0.89	1.12		
	Post-test	C	B-MEC	(Constant)	2.05	1.23		1.67			1.87	0.08
				V-MEM	0.53	0.22	0.28	2.38^*^	1	1		
		E	B-MEC	(Constant)	3.04	0.67		4.52^***^			1.16	0.11
				V-MEIV	0.37	0.14	0.33	2.77^**^	1	1		
			B-MEE	(Constant)	3.01	0.74		4.06^***^			1.42	0.09
				V-MEIV	0.38	0.15	0.30	2.56^*^	1	1		
			B-MEIH	(Constant)	3.66	0.55		6.71^***^			1.12	0.08
				V-MEIH	0.27	0.11	0.29	2.42^*^	1	1		
2 (*n* = 68)	Pre-test	C	B-MEC	(Constant)	3.00	0.57		5.28^***^			2.13	0.08
				V-MEIH	0.30	0.12	0.28	2.40^*^	1	1		
			B-MEE	(Constant)	3.55	0.45		7.83^***^			1.15	0.06
				V-MEE	0.25	0.12	0.25	2.13^*^	1	1		
			B-MEIH	(Constant)	2.64	0.74		3.57^**^			2.15	0.07
				V-MEE	0.36	0.16	0.27	0.28^*^	1	1		
		E	B-MEC	(Constant)	2.56	0.54		4.79^***^			0.37	0.14
				V-MEC	0.30	0.13	0.26	2.27^*^	0.99	1.02		
				V-MEIH	0.25	0.13	0.24	2.03^*^	0.99	1.02		
	Post-test	C	B-MEC	(Constant)	3.38	0.56		6.08^***^			1.83	0.06
				V-MEM	0.24	0.19	0.24	2.05^*^	1	1		
			B-MEIH	(Constant)	1.51	0.74		2.04^*^			2.06	0.23
				V-MEC	0.39	0.12	0.37	3.37^**^	0.98	1.02		
				V-MEM	0.33	0.15	0.25	2.27^*^	0.98	0.02		
		E	B-MEC	(Constant)	1.52	0.68		2.24^*^			0.80	0.24
				V-MEIV	0.38	0.12	0.35	3.09^**^	0.93	1.08		
				V-MEM	0.30	0.10	0.35	3.08^**^	0.93	1.08		
				V-MEE	0.28	0.16	0.27	2.43^*^	0.98	1.03		
			B-MEIH	(Constant)	3.47	0.41		8.45^**^			0.95	0.06
				V-MEIH	0.24	0.12	0.24	2.01^*^	1	1		

*p < 0.05, **p < 0.01, ***p < 0.001. Grouping: 1 = ethnic closeness group, 2 = ethnic alienation group. G, grouping; BI, behavioral intentions; P, priming tasks; C, counterfactual priming; E, empirical priming; DV, Dependent variable; IV, Independent variable; USC, unstandardized coefficients; SC, Standardized coefficients; CS, collinearity statistics; T, Tolerance; DW, durbin-Watson. The initial letter V of the subscale name means minority ethnic value, and B means ethnic behavioral intentions. MEC, minority ethnic consciousness; MEE, minority ethnic exploration; MEIH, minority ethnic inheritance; MEM, minority ethnic mastery.

Another similar test of consistency was conducted in Marks et al. (2014), which was followed in the present study. We separately conducted Pearson’s correlation tests to assess the link between the rating scores of ethnic behavioral intentions in pre- and post-tests (i.e., two blocks of identical trial) of two priming conditions for the closeness and alienation groups of participants. The results revealed that the correlation in MEC, MEE, and MEIH dimensions for the ethnic closeness group was 0.69, 0.52, and 0.40 (*p*_s_ < 0.01) under the counterfactual priming, and was 0.71, 0.60, and 0.80 (*p*_s_ < 0.01) under the empirical priming. For the alienation group, the correlation in the three dimensions was 0.76, 0.81, and 0.73 (*p*_s_ < 0.01) under the counterfactual priming condition, and was 0.83, 0.86, and 0.85 (*p*_s_ < 0.01) under the empirical condition. The correlations of rating scores between the pre- and post-test in the empirical priming condition were higher than that in the counterfactual priming for both groups of participants.

## 4 Discussion

This was the first study to integrate ethnic elements with the sequential priming paradigm. The test program combined commonly used self-reported scores and reaction time as dependent variable indicators and employed counterfactual thinking to explore its impact on consistency between ethnic minority values and value-expressive behaviors. This was reflected not only in behavioral intention scores but also in the reaction time.

### 4.1 The effect of counterfactual treatment on minority ethnic behavioral intentions

Participants with high and low minority ethnic value levels scored higher and responded faster regarding ethnic behavioral intentions in the post-test than in the pre-test under the counterfactual priming condition. This indicated that counterfactual thinking affected ethnic behavioral intention; specifically, it demonstrated a promoting effect. This suggests that counterfactual thinking can psychologically change individuals’ behavioral intentions and promote positive behavioral choices in the future. This finding is consistent with previous studies (Epstude and Roese, 2008; Smallman and Roese, 2009) and appears among a participant group composed of 23 ethnic minorities characterized by their cultural elements in the present study. This confirms the content validity of minority ethnic values as important common reflections of ethnicity and of minority ethnic value-expressive behavior as *a priori* behavior to express their ethnic values (Schwartz and Butenko, 2014; Yang et al., 2019b). Moreover, this validates counterfactual thinking as a measurement indicator of situational effects and extends the method validity to the Chinese ethnic cultural context. This result supported Hypothesis 1 and was accidentally evident among participants in the ethnic alienation group. This may also imply when a minority individual has an ethnic affiliation, and their initial self-construal includes the expectations of their ethnic group, namely ethnicity, which has been found to be important for self-concept (Verkuyten, 2005). A strong environmental threat may increase the bond of ethnicity among ethnic members through engaging in ethnicity-expressive behaviors (Frisch et al., 2014; Cheon and Hong, 2016). Thus, the ethnic minority values, internal values characterized by the importance of ethnicity, of the participants in the ethnic alienation group may have been activated in a situation threatening ingroup identity. This would yield more value-expressive behavioral intentions consistent with those ethnic values than before activation, despite their daily explicit behaviors being alienated from their ethnic characteristics (Schwartz, 1994; Sun et al., 2019). This may be the protective and stimulating effect of ethnicity. The present study also provided a rare empirical evidence on the promoting role of content-specific counterfactuals on ethnic behavior.

### 4.2 The minority ethnic value–behavior intention consistency

Our study showed that the rating scores for behavioral intention in the ethnic closeness group were significantly higher than those in the ethnic alienation group in the pre-test, and even higher in the post-test under both the priming conditions. Furthermore, a significant main effect of ethnic value grouping was observed on the MEE and MEIH dimensions, with a marginally significant effect on the MEC dimension. This implied that higher ethnic value level was associated with more frequently engaging in ethnic value-expressive behavioral intention and the value–behavior intention relationship was stronger at higher level of the relative priority of a specific value (Schwartz, 1992; Lee et al., 2022; Lake et al., 2024). According to description of the value–behavior consistency, the participants in ethnic closeness group engaged daily in the expected behaviors of their ethnic group, which conformed to the motivational goal of their ethnic leading values. Thus, their ethnic values and behavioral intentions were considered consistent. The ethnic alienation group experienced a higher degree of self-perceived alienation from their ethnic group and was associated with being farther psychologically and practically from their ethnic group, being less conscious of their ethnic identity, rarely seeking opportunities to explore their ethnic knowledge, rarely emphasizing the importance of their ethnicity, and rarely engaging in ethnic behaviors (Wan and Wang, 2004; Yang et al., 2019b). Thus, they responded to the degree of agreement and possibility of ethnic behavioral intentions in the test consistent with their low level of minority ethnic values, and their ethnic values and behavioral intentions also presented consistent in the study. These results validate a new pragmatic utility of cognitive psychology, indicating that cognition is enactive and lies in guiding individuals to act effectively (Engel et al., 2016; Ye, 2020). This reaffirms the value theory with ethnic behavior as *a priori* selection of the value expression of ethnic minorities (Schwartz and Butenko, 2014). Moreover, these findings enrich the self-consistency theory (Sheldon and Elliot, 1999; Sheldon et al., 2004) with evidence from the Chinese ethnic culture, in which ethnicity may play the role of a bond. Therefore, Hypothesis 2 was supported.

### 4.3 The effect of counterfactual treatment on minority ethnic value–behavior intention consistency

Although the ethnic closeness group exhibited more significant and positive ethnic behavioral intentions pre- and post-test than the ethnic alienation group in both the paired and independent samples *t*-tests in the counterfactual priming, the expected stronger correlation between ethnic values and behavioral intentions did not emerge pre- and post-test under the counterfactual priming, but did under the empirical priming. The reaction time of the ethnic closeness group was not significantly shorter than that of the ethnic alienation group in either test, but was longer in the pre-test under two priming conditions. These results seem inconsistent with the suggestion of value–behavior consistency (Bardi and Schwartz, 2003) and expected validity of behavioral intention as a measurement indicator for predicting future behavior (Bassili, 1995; Fan and Zhang, 2007; Lewis et al., 2007; Cooke and Sheeran, 2013). In other words, minority ethnic members with a high level of ethnic values should have a stronger statistical relationship with ethnic value-expressive behavioral intentions and respond faster to behavioral intentions expressing their values. Additionally, no significant interaction was found between ethnic value grouping and priming condition across the MEC, MEE, and MEIH dimensions, nor was there a significant main effect of priming condition on the MEC and MEE dimensions. We propose several explanations of these exceptions.

First, under the counterfactual priming condition, the thinking materials used in both tests might have played a role in the result of the value–behavior intention correlation for the ethnic closeness group. The factual thinking materials used in the pre-test were altered descriptions of ethnic alienation behaviors from the dimensions of MEC, MEE, and MEIH of the CMEVEBQ. These are the complete opposites of the daily ethnic value-expressive behavior of individuals with ethnic closeness; thus, they were familiar and common to the ethnic alienation group but unfamiliar and less common to the ethnic closeness group. In the post-test, the descriptions in counterfactual thinking material were some positive ethnic behaviors that were frequently expressed by the ethnic closeness group of participants in daily life. The test program required them to imagine engaging in behaviors in ethnic-value-consistent directions more than usual, which were reversible premises contained in the counterfactual thinking. This placed higher demands on the individuals’ usual high-frequency ethnic behaviors and was another unfamiliar context that differed from daily life. As such, because of unfamiliarity, the thinking materials in either test under the counterfactual priming are not typical behavioral instantiations of minority ethnic values for participants in the ethnic closeness group. However, the descriptions in empirical thinking material were some common, typical, and familiar behavioral intentions for the ethnic closeness group of participants in daily life. They rated ethnic descriptions based on the test program and maintained more consistent minority ethnic value–behavior intention relationships, although their rating scores in the post-test tasks were lower than that under the counterfactual priming. The significant value-behavior intention correlations of MEIV suggested that the ethnic closeness participants’ higher emotional involvement in their own ethnic groups in the familiar situations under empirical thinking priming. The results enriched Konty’s (2002) suggestion regarding value stability in a similar specific environment, validated the value–behavior consistency theory (Verplanken and Holland, 2002; Bardi and Schwartz, 2003) in ethnicity terms, and supported that counterfactual thinking promoted positive behavioral intention (e.g., Smallman and Roese, 2009). Thus, the typicality of value instantiations can help explain why some behavioral intentions are more strongly associated with values than others. Maio et al. (2009a) found that contemplation of typical examples of a value increased subsequent value-related behavior more than the contemplation of atypical examples. Based on this finding, Maio (2010) indicated that value instantiations could affect a strength-related property of the abstract value itself (e.g., value certainty) (p. 27). Lee et al. (2022) listed empirical study results, even across real social groups, which supported the statement that, in social contexts, this can lead to greater effects of attitudes toward a category on relevant judgments and behavior when people are considering typical instances of the category than when considering atypical instances (Chapter 8). Typicality effects are found to be pervasive in the human conceptual system. It may explain why the effect of priming condition (counterfactual vs. empirical) did not differ substantially as a function of an individual’s ethnic value level. Thus, in the current study, as shown in the combined results of correlation and covariance, it may have been the unfamiliar situations induced by the atypical ethnic behavioral instance pre- and post-priming under the counterfactual thinking that triggered more ethnic alienation, which resulted in the absence of anticipated stronger correlations specifically in the ethnic closeness group, with no significant interaction between ethnic value grouping and priming condition.

Moreover, for participants in the ethnic closeness group, the factual thinking materials in the pre-test were certain altered negative ethnic behavioral descriptions. Previous studies suggest that individuals tend to be biased in recognizing negative information; that is, they are more sensitive to negative information (Rozin and Royzman, 2001; Huang and Luo, 2009). Given the same cognitive resources, the activation level of negative information is higher, and deeper processing occurs in the coding and extraction stages (Yongze et al., 2014). This could explain the results indicating that the ethnic closeness group did not respond faster than the ethnic alienation group in the pre-test for the two priming tasks; rather, the former required a longer time to recognize these unfamiliar materials due to the significant amount of cognitive resources (Gawronski and Bodenhausen, 2006). In the post-test, when counterfactual thinking or psychological simulation required the participants in the ethnic closeness group (the high-frequency ethnic behavior individuals) to imagine engaging in behaviors in more ethnic-value-consistent directions than usual, the unreasonableness of this task may have caused negative emotions and greater psychological distress among individuals than the absence of such reactions and thinking (Davis and Lehman, 1995; Roese, 1997). Individuals with ethnic closeness cannot express ethnic behavioral intentions as usual based on their identifiers with strong ethnicity (Verkuyten, 2006), which is the negative function of counterfactual thinking (Markowitz, 2005; Gilbar and Hevroni, 2007; Wei et al., 2009). The reversible conditions may place pressure on individuals’ cognitive resources, resulting in a lower working memory capacity (Bullens et al., 2011; Li et al., 2022). Additionally, due to the effects of the negative information in the pre-test (factual thinking baseline), the ethnic closeness participants may require a longer time in the post-test to adapt themselves back to rate ethnic behavioral intentions (Gawronski and Bodenhausen, 2006; Yongze et al., 2014), thus they still did not respond faster even under their familiar empirical thinking. A marginal significance was observed between the value–behavior intention correlation of MEE (p_pre_ = 0.05, p_pro_ = 0.05) and a significance for MEM (*p* < 0.05) in the post-test, suggesting a tendency for their exploration and adaptation intentions (Yang et al., 2019b) to increase more in the post-test than in the pre-test. Thus, for the ethnic closeness group, both the negative information context in the pre-test of factual thinking and the counterfactual context that may challenge ethnicity in the post-test could be realistic obstacles, namely the “reality” described by Shoda (1999), when it comes to making the optimal choice in a value-consistent direction, building a strong and expected minority ethnic values–behavior intention relationship, and demonstrating a significant main effect of priming condition on both behavioral intention and reaction time.

Second, for the ethnic alienation group pre- and post-test, an improving value–behavior intention correlation emerged in the MEC dimension of two priming conditions, which refers to minority ethnic self-awareness of own ethnic affiliation and matters related to own ethnic group (Yang et al., 2019b). Before starting the test tasks, we checked the participants’ ID cards to confirm that they were minority ethnic members. Then, through the combined influence of factual thinking materials related to ethnic behavioral intention descriptions, reflections on ethnic alienation triggered by materials for thinking priming, and the behavior-intention judgments required by the tests, participants in the ethnic alienation group may have gradually developed a heightened awareness of their minority ethnic identity. This empirically tests Verkuyten (2005) suggestion that the fading cultural differences may particularly cause the most acculturated minority individuals to emphasize their ethnic belonging. MEIH value correlated most strongly with its corresponding behavioral intentions in the post-test of two priming conditions, which suggesting that the ethnic alienation participants made behavior-intention choices consistent with values despite the influence of various factors. In the study, the counterfactual priming tasks asked ethnic alienation participants to change from their adapted and familiar ethnic contexts (pre-test), moving away from ethnicity, and to fit into the new contexts (post-test) closed to ethnicity; whereas the thinking materials of empirical priming were some high-frequency ethnic behavioral descriptions and also negative information for participants in the ethnic alienation (Rozin and Royzman, 2001; Huang and Luo, 2009). Driven by heightened ethnic consciousness, they maintained robust and consistent value–behavior intention correlations in the MEIH dimension, with the strongest correlation being between MEC value and MEIH behavioral intentions in the post-test of counterfactual priming, and between MEIH value and MEC behavioral intentions in the pre-test across both priming conditions.

In addition, according to Construal Level Theory, the predictive power of values on behavioral intention is contingent on the level of construal that these values elicit for the given situation, and temporal changes in construal can influence an individual’s choice preferences (Liberman et al., 2007; Trope and Liberman, 2003). Specifically, with greater temporal distance, individuals’ attention shifts away from feasibility (i.e., the means to achieve that end-state) and toward necessity (i.e., values concerning the end-state of an action) (Sagristano et al., 2002). In the current study, the ethnic alienation group rarely engaged in ethnic exploration and inheritance and sought the corresponding opportunities and means, thus their MEE and MEIH values (the future activities) correlated most strongly with the ethnic behavioral intentions of MEC value (an end-state of ethnicity), MEIH value explained the most variance of the ethnic behavioral intention of MEC in the pre-test of both the priming tasks, and the significant main effect of the priming condition was restricted to the MEIH dimension, with no significant effects found on the MEC or MEE dimensions. This also supported Yang (2002) empirical findings that near-future ethnic exploration served to promote distant-future ethnic behaviors, e.g., ethnic inheritance, a process that is itself sustained by increased engagement in ethnic exploration in the present, with MEE value correlating most strongly with the behavioral intention of MEIH under counterfactual treatment. These results showed that ethnic exploration promotes ethnic inheritance and that ethnic inheritance reinforces ethnic consciousness, which also supported the suggestions that value stability in similar specific environments (Konty, 2002) and typical examples of values (Hanel et al., 2018) can promote behaviors congruent with values.

Third, a significant correlation was observed between MEM value and the behavioral intentions of MEC for two groups of participants under both the counterfactual-empirical thinking priming. MEM as a cultural value refers to the relationship between an ethnic minority and society and nature (Schwartz, 2014; Yang et al., 2019b). Mastery encourages active self-assertion to attain group or personal goals and emphasizes changing to adapt to the social and natural world rather than fitting into it or acceptance. In the current study, participants with various levels of ethnic minority values were involved in different ethnic contexts triggered separately by factual, counterfactual, and empirical thinking. Ethnicity for minority ethnic groups requires them to adapt to new social environments, such as the situations of psychological simulation in this study, which promotes ethnic closeness participants’ self-awareness of own ethnic affiliation and matters related to own ethnic group with the most variance (*β* = 0.53) of the post-test behavioral intention score of MEC appearing in the value of MEM under the counterfactual priming. This trend was especially obvious for ethnically alienated individuals, with the correlations between MEM value and the ethnic behavioral intentions of MEC, MEE, and MEIH being higher than that among the ethnic closeness group in each thinking task and the predictor value in MEM explaining the most variance of MEC (*β* = 0.24) behavioral intention scores after the counterfactual priming. Before priming tasks (factual thinking baseline), individuals with ethnic alienation faithfully fulfilled role obligations as ethnic members and persistently struggled to master “reality” by changing, directing, and exploiting to attain personal goals of ethnic inheritance, just as they are almost fully integrated into mainstream culture in real life. After counterfactual priming tasks, however, they attempted to adapt themselves to ethnic environments, such as mental simulation contexts constructed from ethnic scenarios, through a heightened ethnic awareness but failed to fit into those environments to attain group goals of ethnic inheritance. Moreover, participants in the ethnic closeness group demonstrated greater adaptive change under the counterfactual treatment that challenged ethnicity than those in the ethnic alienation group. This suggested that importance of ethnicity protected and motivated minority ethnic individuals to maintain consistency between ethnic values and value-expressive behavioral intentions. The underlying process likely attenuated the substantial difference of priming condition as a function of an individual’s ethnic value level, with no significant interaction between ethnic value grouping and priming condition. The results provide new statistical evidence on how ethnic minority populations develop mastery in adapting to new social environments.

Therefore, the counterfactual thinking that implies the importance of ethnicity, as a specific situational trigger, affected the degree of consistency of minority ethnic values with the value-expressive behavioral intentions of the two groups of participants. This manifested as implicit and explicit ethnic value–behavior intention consistency in the ethnic closeness and alienation groups, respectively. The results shown in correlation, regression, and covariance validated the value–behavior intention consistency theory (Verplanken and Holland, 2002; Bardi and Schwartz, 2003) in ethnicity terms and supported Hypotheses 2 and 3.

## 5 Conclusion and implication

### 5.1 Conclusion

This study used a sequential priming paradigm to explore how counterfactual treatment affected the ethnic behavior-intention expression of individuals with different values levels and minority ethnic value–behavior intention consistency. The results indicated that, compared to members with low minority ethnic value levels, individuals with high minority ethnic values more strongly expressed their ethnic behavioral intention in ethnic-value-consistent directions in both counterfactual and empirical (familiar) situations. In addition, under different effects (inhibiting and promoting) of potential situational constraints, minority ethnic value–behavior intention consistency was more conflicted and implicit at lower and significant at higher ethnic-minority-value level. This reflects a bond of ethnicity.

### 5.2 Research implications

Although most studies have suggested non-negligible effects of situational factors on value–behavior relationship, evidence on the promotion or inhibition of value–behavior consistency by factor content, influential mechanisms, and situation manipulation is limited. This study extends the research on value–behavior-consistent relationship among minority ethnic populations, provides psychometric evidence triggered by counterfactual thinking, and examines the relationship and mechanisms between minority ethnic values and their value-expressive behavioral intentions under counterfactual treatment based on self-construal and value theory and operational definition of minority ethnic values in the context of ethnic psychology. The results enrich the research on ethnic populations in the field of ethnic psychology, verify the bond effect of ethnicity on the consistency of minority values and behavioral intentions, and provide a rare empirical basis and suggestions for ethnic consciousness elevation and ethnic behavior promotion.

First, Chinese national ethnicity was formed in the process of the joint creation of a Chinese civilization and forging of the Chinese nation by the people of all ethnic groups in China since ancient times. This is also a practical example of national integration of various minority ethnic groups through ethnic mastery (MEM). At present, projects focusing on inheriting and developing the Chinese civilization and studying and exploring its inherent national ethnicity and contemporary values serve as a crucial method to enhance people of all ethnic groups to identify themselves with a community for the Chinese nation, thereby facilitating their national value–behavior intention consistency (Shechter et al., 2011). This conforms to the results of this study that ethnic exploration promotes ethnic inheritance and that ethnic inheritance reinforces ethnic consciousness. For example, by integrating national major public events (e.g., the 2008 Beijing Olympics) into social-cultural practice to awaken the collective memory of the Chinese nation among people of all ethnic groups (Chen and Zhang, 2023), such situational priming of community for Chinese nation can stimulate ethnic minorities’ sense of oneness (Long et al., 2025). Moreover, the past time orientation promotes the sense of the Chinese nation community among ethnic minorities (Guan and Zhou, 2024). Second, the varying degrees of impact of counterfactual treatment on the consistency of ethnic values and behavioral intentions among individuals with high-low levels of ethnic minority values suggests greater efforts to exert national closeness group among the Chinese nation on building an interconnected social structure and community environment and promoting communication, exchanges, and integrations among people from different ethnic groups. This can promote national alienation individuals to fit into Chinese national environments. For example, the construction of a demonstration area for forging a stronger consciousness of the Chinese national community (Ling, 2023), where people of different ethnic groups live together, can, through frequent intergroup contact, enhance the understanding of various ethnic groups when it comes to the languages, cultures, customs, and habits of other ethnic groups (Dovidio et al., 2017) while also effectively reducing intergroup prejudices and negative stereotypes (Brochu et al., 2020).

### 5.3 Limitations and future directions

Despite the interesting findings, this study has several limitations that should be considered. First, our study recruited participants among minority ethnic university students. Our participants required at least middle education to complete the survey and test independently, and this excluded the ethnic minorities who had poor education levels but may have had strong self-perceived ethnicity (ethnic minority values); moreover, the relative homogeneousness of the participants and 27% extreme-grouping naturally limits the broader applicability of the present study; thus, future studies should consider more population factors, such as diverse occupations mixed with different educational stages (middle, high, and vocational school) and other Chinese Minzu universities, as well as minority gathered-residing regions, so as to broaden the applicability of these research results. Second, the use of a within-subjects design with a fixed order of priming conditions introduces a threat to internal validity due to potential order effects (e.g., carryover or fatigue). Although this design was chosen to control for individual differences and increase statistical power with a limited sample size, the observed differences between the factual and counterfactual (empirical) priming conditions could be partially attributed to the sequence of presentation. Future research should employ either a fully counterbalanced within-subjects design or, ideally, a between-subject manipulation of the priming variable with random assignment. Third, we selected items contents as the ethnic behavioral materials from 3 of 6 dimensions of the CMEVEBQ, and each dimension only contained one or two cases, which may affect the degrees of consistency of value–behavior intention relations due to the lack of typical behavioral instantiations of expressing values. Future research may seek more typical ethnic behaviors expressing ethnic values and alter them to the corresponding factual, counterfactual, and empirical thinking materials to obtain more evidence of ethnic value–behavior consistency. Finally, as behavioral intentions cannot provide an accurate understanding of how minority ethnic values are associated with value-expressive behaviors; thus, replication is required to explore behaviors in the real world as well as in laboratories. Furthermore, future research should explore other situational variables that associate ethnic values and corresponding behaviors.

## 6 Attachment

North Minzu University, under the direct administration of the National Ethnic Affairs Commission of the People’s Republic of China, is a comprehensive institution of higher learning specialized in ethnic minorities affairs. It is located in Yinchuan, the capital city of Ningxia Hui Autonomous Region and is the only university established in minority autonomous region in China under the direct administration of the Commission. The university now has more than 21,000 postgraduates, undergraduates and preparatory students from 56 ethnic groups, including 55 officially defined ethnic minority groups and one majority group (ethnic Han), in 31 provinces, municipalities and autonomous regions. The proportion of ethnic minority students is 60%, and their population information, such as ethnic diversity, residence status (e.g., gathered-residing and scattered-residing), and source area (e.g., urban and rural), conforms to minority ethnic characteristics in China, which is the microcosm of the Chinese nation.

## Data Availability

The raw data supporting the conclusions of this article will be made available by the authors, without undue reservation.
